# Efficient System Reliability-Based Design Optimization Study for Replaced Hip Prosthesis Using New Optimized Anisotropic Bone Formulations

**DOI:** 10.3390/ma13020362

**Published:** 2020-01-13

**Authors:** Ghais Kharmanda, Samer Gowid, Elsadig Mahdi, Abdallah Shokry

**Affiliations:** 1 Mechanics Laboratory of Normandy, INSA Rouen, 76801 St Etienne du Rouvray, France; mgk@scs-net.org; 2 Mechanical and Industrial Engineering Department, Qatar University, Doha 2713, Qatar; Samer@qu.edu.qa (S.G.); elsadigms@qu.edu.qa (E.M.); 3 Department of Mechanical Engineering, Fayoum University, Fayoum 63514, Egypt

**Keywords:** hip prosthesis, bone material properties, structural reliability, reliability-based design optimization

## Abstract

An efficient reliability algorithm is developed to transfer the system reliability problem to a single-component reliability problem, considering the uncertainty of loading cases and the material properties. The main difficulty is that femoral bone densities change after hip arthroplasty and, thus, the mechanical properties of the distinctive bone tissues and, therefore, the corresponding elasticity modulus and yield stress values change. Therefore, taking these changes into account during the hip prosthesis design process is strongly needed. As the bone possesses anisotropic behaviors, as the material properties in both radial and tangential directions in long bone (femur, tibia) are almost similar, the bone anisotropy is represented in this study by transversal isotropy. Two optimized formulations for yield stress against the elasticity modulus relationship are first developed and then integrated into an efficient reliability algorithm. Thus, a coupling between reliability and optimization, so-called reliability-based design optimization (RBDO), is introduced in order to control the reliability level. The proposed RBDO algorithm using optimum safety factors (OSF) takes into account the material uncertainties and leads to new stem dimensions. An in-depth numerical analysis on a cementless hip prosthesis is implemented to demonstrate the appropriateness of the proposed algorithm with the consideration of many different loading cases. The results show that the studied model can be effectively used when compared to previous works, which concerns the changes in both geometry and material properties.

## 1. Introduction

Hip arthroplasty is an innovative subject in orthopedic surgery, as it permits a significant advancement in pain and functional capabilities, in addition to a high patient justification level with the embedded prosthesis. The primary goal of this process is the long-time continuation of the implant while maintaining steady obsession of the parts with negligible wear of the contact surfaces [1]. The introduction of reliability and failure probability concepts can play an important role in this context [2,3]. Cemented and cementless stem implantation results in considerable changes in femoral load transfer, in which the femoral bone responds to these changes with a process known as versatile bone remodeling that is controlled by Wolff’s law [4].

As a matter of fact, bone remodeling relies on mechanical and biological elements [5]. The mechanical part is associated with stress distribution enforced by the femoral stem, as well as implant features, i.e., size, shape, alloy, and also the kind of bone fixation [2,6,7,8,9,10,11]. When using the deterministic optimization strategies for prosthesis design, empirical safety factors can be proposed without including the effects of uncertainties concerning materials, geometry, and loading. This way, the optimized configuration may present a lower reliability level and may lead to a higher failure rate. A reliability analysis can be carried out in order to assess the reliability level of the resulting prosthesis design [1]. Here, the purpose of the reliability analysis is to evaluate the reliability levels in the structural design stages—taking the uncertainties of the material properties and loading into consideration.

On the one hand, the living tissue modeling difficulty is related to the change in the loading and bone material properties after prosthesis arthroplasty. Considering bone as an anisotropic material, the elastic and strength properties in the axial direction are different than those in the radial direction, in which the bone is found to have higher strength values when applied in the diaphyseal axis (longitudinal direction) compared to those values obtained in the radial or circumferential “transverse” directions. Relatively, the variations in the computed modulus and strength in the radial and circumferential directions have been found to be small, so human bone can be considered and modeled as transversely isotropic. A possible reason is that the growth and adaptation of the bone is most efficiently related to the uniaxial stresses that develop along the diaphyseal axis during habitual activities, such as gait [12]. These properties are correlated to the bone densities, which change after a prosthesis arthroplasty, and must be treated as orthotropic materials in certain cases. In brief, cortical and cancellous bone are treated as anisotropic materials, while the stem and the ball are considered isotropic materials due to the fact that their properties remain unchanged after prosthesis arthroplasty.

In addition to that, the prosthesis design is considered a large-scale optimization problem due to the consideration of a large number of constraints and variables. A reliability analysis of such a large problem should be performed using a particular optimization procedure [13], and it is compulsory to develop efficient computational algorithms to solve it. The classical algorithms such as Rackwitz–Fiessler consider a single failure mode [14]. These algorithms employ gradient optimization methods and, hence, a single search direction only is required to minimize the objective function [13]. Therefore, the multiple failure case necessitates dealing with several optimization problems, where each of these problems has a single failure mode only. However, in this work, the non-gradient optimization method (zero-order method), so-called curve fitting method, is used to overcome this drawback. Thus, the optimization process is performed according to an approximation technique. In this study, the integration of the reliability concept [14] is carried out considering the variation effect of the limit state functions.

This way, a direct assessment may lead to an unsuitable reliability level; therefore, it is very important to use the concept of the reliability-based design optimization (RBDO). In Kharmanda et al. [15], a RBDO algorithm based on the hybrid method has been elaborated to integrate the RBDO model into the orthopedic prosthesis design such as hip design. In this case, the integration of the hybrid method leads to a high computing time because the total number of optimization variables is the sum of the design variables and the random variables. In this work, the optimum safety factors (OSF) are integrated to reduce the computing time and simplify the RBDO process.

In summary, for the hip prosthesis design process, there is a considerable need to take the changes of the material properties of the different bone tissues and the corresponding elasticity modulus and yield stress values into account. On the other hand, some muscles can be cut or harmed during surgery operation, and consequently, they cannot operate at their maximum capacity. Accordingly, it is important to introduce the uncertainty to the loading during the prosthesis design.

In this study, optimized formulations for yield stress against the elasticity modulus relationship are developed to dynamically consider the changes in the material properties. The uncertainty to loading cases is studied in order to take the maximum loading capacity of the joint into account. In addition, an integrated reliability model that considers the variation effect of the limit state functions is developed to provide an integrated design solution to the addressed problem.

## 2. Models and Methods

### 2.1. Studied Design

The developed reliability strategy is applied to the hip prosthesis design. The main difference between the previous structural reliability method and current strategy is the integration of living tissues, where bone continues to remodel after the replaced hip implantation [1]. The age of the patient affects the bone remodeling process, which leads to a complicated reliability assessment, given that bone has been assumed to be isotropic in a number of previous research articles [2,15]. However, bone is an anisotropic material. The studied case (long bone) is considered a special anisotropic case as small differences in modulus and strength have been reported between the radial and circumferential directions, indicating that human cortical bone may be treated as transversely isotropic. The assumption of transversal isotropic bone behavior allows a 2D model to be used during the reliability assessment and also leads to a reduction in the computing time. In addition, the limit state functions vary during the reliability analysis. Thus, two optimization formulations are integrated into the developed reliability algorithm.

#### 2.1.1. Geometry Description

**Figure 1:**
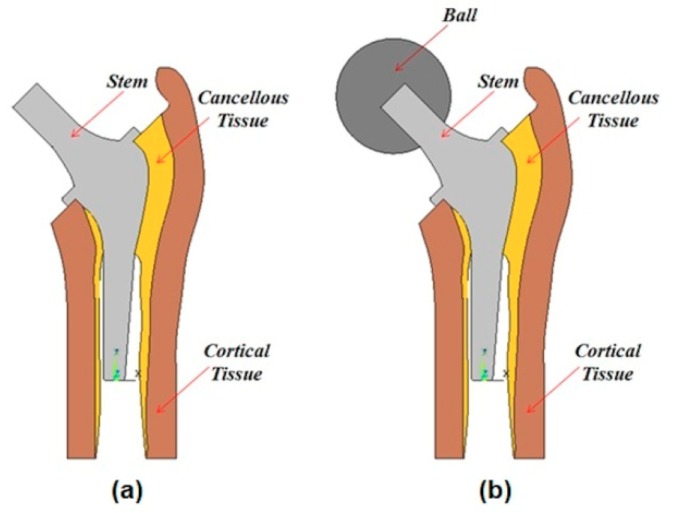
Illustration of the different parts of the hip prosthesis: (**a**) The previous model [1], and (**b**) the newly proposed model.

A 2D finite element model of the studied cementless hip prosthesis with different parts is shown in Figure 1. In the previous published work by Kharmanda [2], the study was carried out without considering the effect of the ball during the optimization process to take the applied force position into account (see Figure 1a). Furthermore, the marrow layer was considered as a cavity and then neglected because it was assumed to have a minor influence on the mechanical response [16]. In addition, the density of the femoral bone has been found to change after hip arthroplasty with different forms of stems [1]. The previous studies did not take the integration of uncertainty concepts during the design process into consideration, in which the obtained results can be affected. Figure 1b shows the newly proposed model taking into account the effect of the ball and considering bone anisotropy.

#### 2.1.2. Boundary Conditions

**Figure 2:**
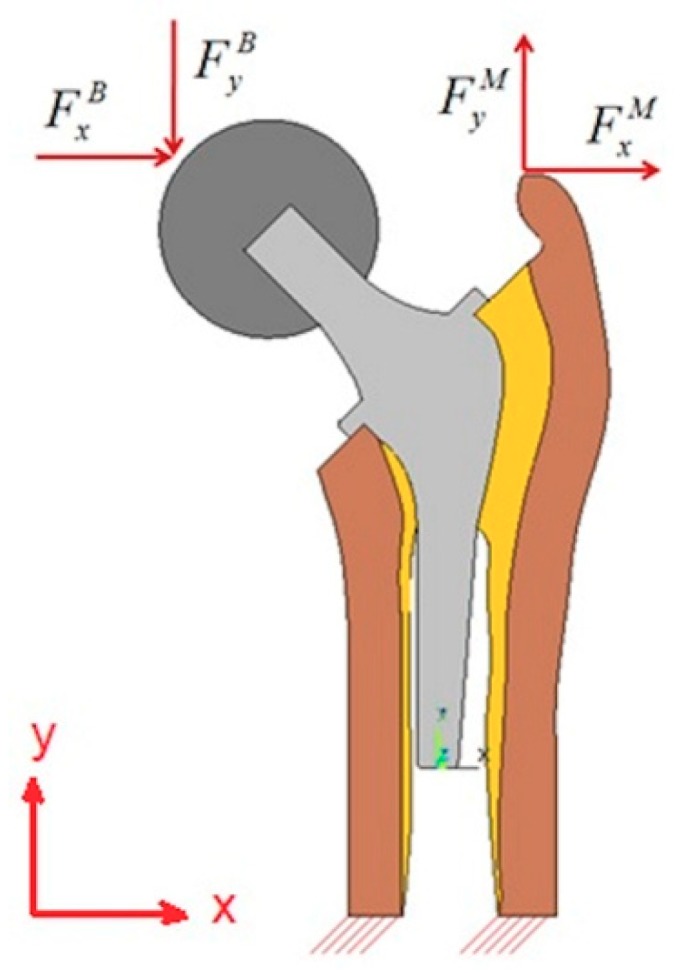
Boundary conditions and applied loads of the newly proposed model.

In this work, three different day-by-day loading cases are implemented: one-legged standing (L1), abduction (L2), and adduction (L3) [17]. Figure 2 shows the newly proposed model with boundary conditions of u_2_ = 0 at the lower cut in the cortical part, and with the following applied loads: The body (F^B^) and muscle forces (F^M^) in the x- and y-directions. The used components of different forces corresponding to the three loading cases: L1, L2, and L3 that are shown in Figure 2 are presented in Table 1.

#### 2.1.3. Meshing and Material Properties

The number of elements used for the presented model is 1542 non-linear elements with element type, 8-node/PLANE82, and with a total number of 5027 nodes. The materials of the ball and the stem are considered homogeneous and isotropic. The number of elements is 255 for the ball and 485 for the stem. The stem is assumed to be made of a titanium-based alloy (Ti-6Al-4V) with Young’s modulus E=110 GPa and Poisson’s ratio ν=0.3, while the ball is assumed to be made of a ceramic alloy (Al_2_O_3_) with Young’s modulus E=408 GPa and Poisson’s ratio ν=0.27. The corresponding yield stress for the stem is 825 MPa and for the ball is 250 MPa [18]. The cortical and cancellous bone parts are assumed to be transversal isotropic materials. The cortical region is modeled with 596 elements, and the cancellous region is modeled with 206 elements. The material properties of the cortical and cancellous tissues are presented in Section 3.1. The marrow region is modeled as a cavity and consequently neglected in the finite element analysis [16].

### 2.2. Developments for Material Properties

In several published works, the yield stress and also the Young’s modulus are heavily connected to bone density. In addition, bone has been modeled as isotropic and homogeneous materials; however, the anisotropy of bone is probably the most critical mechanical characteristic. For example, during the gait cycle, largely uniaxial stresses can be produced along the diaphyseal axis. In the context of bone anisotropy, Lotz et al. [19] computed the Young’s modulus, as well as the compressive strength, of femoral bone for cortical and cancellous in the axial and transversal directions using the apparent density as a control variable. In the axial direction, both modulus and compressive strength for the cortical were approximated by

(1) $$E=2065\rho^{3.09},$$

(2) $$\sigma_{C}=72.4\rho^{1.88},$$

and in the transversal direction, they were correlated as

(3) $$E=2314\rho^{1.57},$$

(4) $$\sigma_{C}=37\rho^{1.51}.$$

Similarly, the elastic modulus and compressive strength for cancellous bone in the axial direction were defined as

(5) $$E=1904\rho^{1.64},$$

(6) $$\sigma_{C}=40.8\rho^{1.89},$$

and in the transversal direction, they were correlated as

(7) $$E=1157\rho^{1.78},$$

(8) $$\sigma_{C}=21.4\rho^{1.37}.$$

Kharmanda [2] used the previous equations, and generalized a formula that correlates the modulus and yield strength with the apparent density, which can be introduced as

(9) $$E=A_{E}\rho^{n_{E}},$$

(10) $$\sigma_{C}=A_{\sigma }\rho^{n_{\sigma }},$$

where $A_{E}$ and $A_{\sigma }$ are two constants, and $n_{E}$ and $n_{\sigma }$ are two exponents. Eliminating $\rho_{\alpha }$ from Equations (9) and (10) leads to

(11) $$\sigma_{C}=A_{\sigma }{\left(\frac{E}{A_{E}}\right)}^{R_{\sigma /E}},$$

where $R_{\sigma /E}=n_{\sigma }/n_{E}$ is the ratio of exponents. According to Lotz’s model, Equation (11) in the cortical bone can be written for the axial direction as

(12) $$\sigma_{C}=72.4{\left(\frac{E}{2065}\right)}^{0.61},$$

and for the transversal direction, it can be given as

(13) $$\sigma_{C}=37{\left(\frac{E}{2314}\right)}^{0.96}.$$

According to Lotz’s model, Equation (11) in the cancellous bone can be written for the axial direction as

(14) $$\sigma_{C}=40.8{\left(\frac{E}{1904}\right)}^{1.15},$$

and for the transversal, it can be given as

(15) $$\sigma_{C}=21.4{\left(\frac{E}{1157}\right)}^{0.77}.$$

To obtain the optimum fitness of Equation (11), at least three experimental points should be considered in order to find the constant values. According to the previous work by Kharmanda [2], the optimized formulation for cortical bone in the axial direction can be expressed as

(16) $$\sigma_{C}=129.5{\left(\frac{E}{11142}\right)}^{0.94}.$$

Accordingly, the three experimental results that are presented in Table 2 are used to describe the optimum fitness in the transversal direction. Consequently, the new optimized formulation can be expressed as

(17) $$\sigma_{C}=125{\left(\frac{E}{8311}\right)}^{0.24}.$$

The optimized formulations (Equations (16) and (17)) for the cortical bone in both axial and transversal directions are compared to Lotz’s model (Equations (12) and (13)). Table 2 and Table 3 show that the optimized formulations lead to high-accuracy results compared to Lotz’s model.

**Figure 3:**
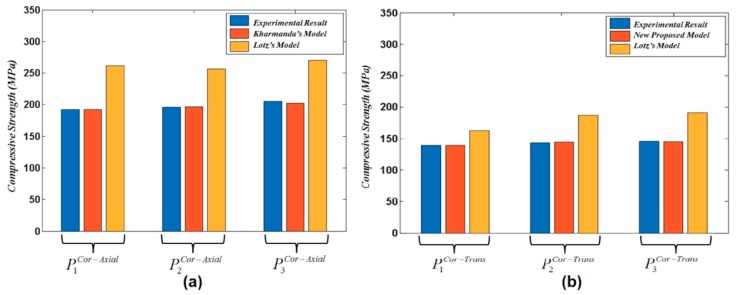
A comparison between experimental compressive stress in cortical bone for (**a**) Lotz’s model (Equation (12)) and Kharmanda’s model (Equation (16)) in the axial direction, and (**b**) Lotz’s model (Equation (13)) and the newly proposed model (Equation (17)) in the transversal direction.

A graphical representation of the compressive stress values that are presented in Table 2 and Table 3 is shown in Figure 3. Figure 3a shows a significant difference between the experimental results and Lotz’s model for cortical bone in the axial direction, while Kharmanda’s model leads to more accurate results. In addition, the newly proposed model in the transversal direction gives compressive stress values that are much closer to the experimental results, and more accurate than those obtained by Lotz’s model, as shown in Figure 3b.

**Figure 4:**
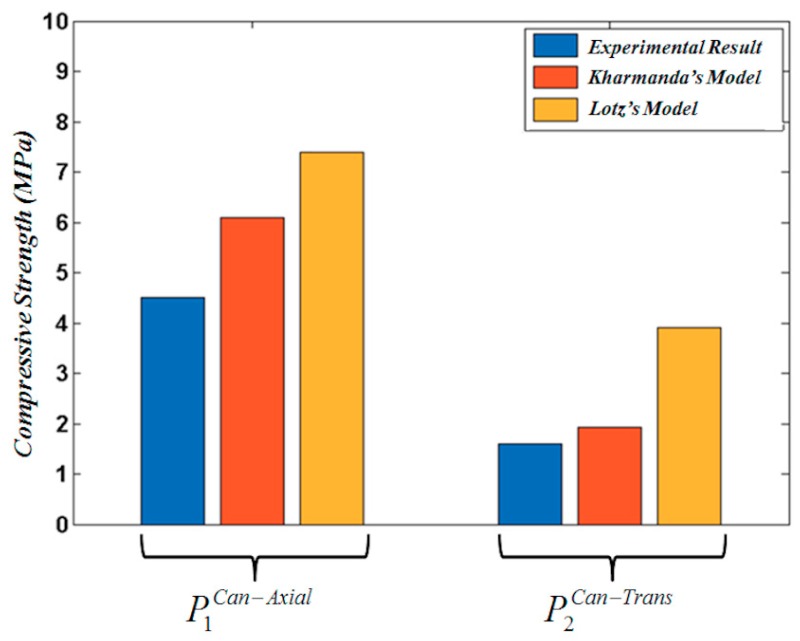
A comparison between experimental compressive stress for Lotz’s model (Equation (14)) and Kharmanda’s model (Equation (16)) for cancellous bone in the axial direction, and Lotz’s model (Equation (15)) and Kharmanda’s model (Equation (16)) for cancellous bone in the transversal direction.

Giesen et al. [22] tested the cancellous bone in both directions for several specimens. A comparison of experimental compressive stress with Lotz’s and the newly proposed models for cancellous bone is tabulated in Table 4 and represented graphically in Figure 4. By means of comparing the optimized formulations (Equations (16) and (17)) for the cancellous bone in both axial and transversal directions with Lotz’s model (Equations (14) and (15)), it is found that the resulting values of the optimized formulation are nearer the experimental results than those calculated by Lotz’s model. It is also interesting to notice that the difference between the experimental and newly proposed model results in the transversal direction is much lower than that obtained between the experimental and Lotz’s model, which means that the newly proposed formulation can be used effectively in the transversal direction considering bone anisotropic material.

The tensile yielding stress can be presented as a fraction of the compression strength for the axial direction, and can thus be written as

(18) $$\sigma_{T}=R_{T/C}.129.5{\left(\frac{E}{11142}\right)}^{0.94},$$

and in the transversal direction, it can be written as

(19) $$\sigma_{T}=R_{T/C}.125{\left(\frac{E}{8311}\right)}^{0.24}.$$

Different values have been used for the ratio $R_{T/C}$, from 0.5 to 0.7 for cortical bone and from 0.7 to 1 for cancellous bone [23].

### 2.3. Developed Reliability Algorithm

The goal is to seek the most probable point (MPP) that is defined by the minimum distance between the source of the normalized space and the most important failure surface (limit state function). Related to the earlier stem-bone simulations, the von-Mises stress at the cancellous region has been found to be the most effective element of the limit state function. In the interest of resolving the reliability issue, for the given failure scenario, the reliability index β is achieved by solving a constrained minimization problem:

(20) $$\begin{array}{l} \min:d(u_{i})=\sqrt{\sum_{i=1}^{n}u_{i}^{2}}\\ s.t.:H(u_{i},y_{i})=0\\ \;\;:g_{1}(u_{i},y_{i})\leq 0\\ \;\;:\ldots \\ \;\;:g_{m}(u_{i},y_{i})\leq 0 \end{array}$$

**Figure 5:**
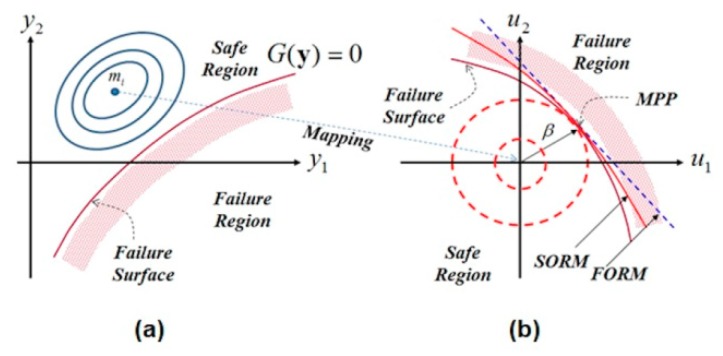
(**a**) Physical space and (**b**) normalized spaces.

Equations (18) and (19) can be integrated into the constraints in Equation (20). According to Hasofer and Lind [24], operating in the standard normalized space of independent Gaussian variables (see Figure 5b) is much better to operating in the space of physical variables (see Figure 5a).

**Figure 6:**
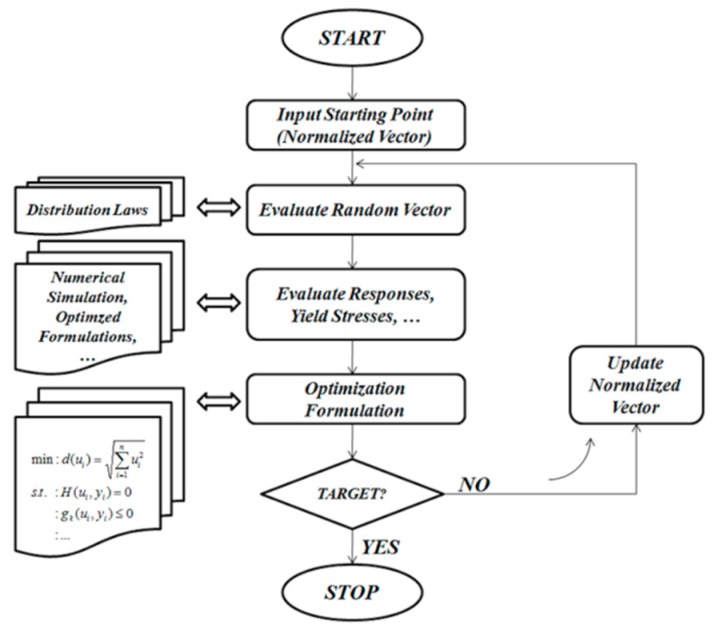
Flowchart of developed reliability algorithm.

Hence, a normalized variable $u_{i}$ can be defined as

(21) $$u_{i}=\frac{y_{i}-m_{i}}{\sigma_{i_{}}},$$

where $y_{i}$ introduces a random variable, $m_{i}$ represents the mean value, and $\sigma_{i}$ represents the standard deviation. $m_{i_{}}$ may be chosen as a design variable $x_{i}$(see Figure 5a). $\sigma_{i}$ is assumed to be proportional to the mean values (50%). The goal and the constraint functions are demonstrated clearly in the optimized algorithm shown in Figure 6. The algorithm minimizes the minimum distance $d(u_{i})$.

### 2.4. Developed RBDO Algorithm

The RBDO algorithm is carried out using the OSF method. The RBDO procedure contains three main steps:

The first step is to obtain the failure point (MPP). The optimization process minimizes an objective function subjected to the design constraint without consideration of the safety factors. This way, the optimization problem for a single failure mode is simply written as

(22) $$\begin{array}{l} \min:\;F\left(y\right)\\ s.t.\;:\;g(y)\leq 0 \end{array}$$

and for multiple failure modes, it is given as follows:

(23) $$\begin{array}{l} \min:\;F\left(y\right)\\ s.t.:\;g_{1}(y)\leq 0\\ \;\vdots \\ \;:\;g_{m}(y)\leq 0 \end{array}$$

The second step is to compute the optimum safety factors. Here, the sensitivity values of the limit state function(s) with respect to all variables are needed. It has been demonstrated that the optimum values of the normalized vector $u_{i}^{*}$ for single failure case can be written as in the following form [25]:

(24) $$u_{i}^{*}=\pm \beta_{t}\sqrt{\frac{\left|\frac{\partial G}{\partial y_{i}}\right|}{\sum_{j=1}^{n}\left|\frac{\partial G}{\partial y_{j}}\right|}},$$

while for the multiple failure case, it can be written as

(25) $$u_{i}^{*}=\pm \beta_{t}\sqrt{\frac{\left|\sum_{j=1}^{m}\frac{\partial G_{j}}{\partial y_{i}}\right|}{\sum_{i=1}^{n}\left|\sum_{j=1}^{m}\frac{\partial G_{j}}{\partial y_{i}}\right|}},$$

where the sign of ± depends on the sign of the derivative. For the normal distribution law, the optimum safety factor is given by

(26) $$S_{f_{i}}=1\pm u_{i}^{*}.$$

The final step is to calculate the optimum solution. This encompasses inclusion of the resulting values of the safety factors into the design variables in order to evaluate the optimum solution.

The efficiency of the OSF method has been demonstrated relative to the hybrid one [26]. Recently, the efficiency of OSF formulations has been demonstrated considering both the reliability index method (RIA) and performance measurement approach (PMA) for normal and lognormal distribution laws [27].

## 3. Results

### 3.1. Reliability Results

**Figure 7:**
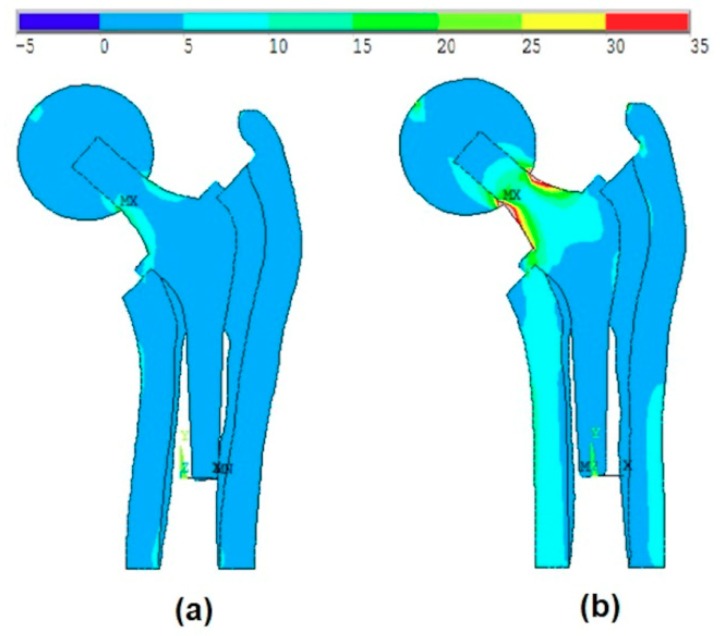
The effect of the introduction of the ball and bone anisotropy on the von-Mises stress distribution at (**a**) the mean point and (**b**) the most probable point (MPP).

The yield stress is considered the limitation of failure for all layers. The failure scenario can occur in each layer. The stem and ball parts are made of isotropic materials and their failure scenarios are $\sigma_{\max}^{Stem}-\sigma_{Yield}^{Stem}\leq 0$ and $\sigma_{\max}^{Ball}-\sigma_{Yield}^{Ball}\leq 0$, respectively. However, the cortical and cancellous tissues are transversal isotropic materials and their failure scenarios are considered in two directions. The failure scenario for the cortical tissue in the transversal and axial directions are $\sigma_{\max}^{Cor-Trans}-\sigma_{Yield}^{Cor-Trans}\leq 0$ and $\sigma_{\max}^{Cor-Axial}-\sigma_{Yield}^{Cor-Axial}\leq 0$, respectively. The failure scenario for the cancellous tissue in the transversal and axial directions are $\sigma_{\max}^{Can-Trans}-\sigma_{Yield}^{Can-Trans}\leq 0$ and $\sigma_{\max}^{Can-Axial}-\sigma_{Yield}^{Can-Axial}\leq 0$, respectively. The problem contains 22 random variables: 4 for the forces and 18 for the material properties (see Table 5). The components of weight and muscle forces for the three daily loading cases are considered (see Figure 2). The presented values in Table 5 are considered as proportional values as the study is carried out in 2D. When evaluating the reliability index of a studied configuration, this studied configuration is statistically represented by the mean point while the algorithm searches the most probable failure point (MPP). The image of the distance between the two configurations (mean point and MPP) in the normalized space (see Figure 5b) is called the reliability index β. According to formulation 21, the mean point is represented by the mean vector $m_{y_{i}}$ of the random vector $y_{i}$. In the previous works [1,15], where the bone isotropy was considered, the maximum von-Mises stress was considered a failure indicator, while in this work, it is calculated for comparison to the other studies and not as a failure indicator. Here, the maximum stress values in the transversal and axial directions should not exceed the yield stress (elasticity limits) in the corresponding direction. These maximum stress values are considered as failure indicators. In the following, the reliability results of the three different loading cases L1, L2, and L3 are explained:

Case 1:

Figure 7a shows the effect of the introduction of the ball and bone anisotropy on the von-Mises stress distribution at the mean point, while Figure 7b shows the von-Mises stress distribution at the MPP.

The MPP is found on the failure scenario of the cancellous tissue in the transversal direction where the reliability index is β=3.91.

**Figure 8:**
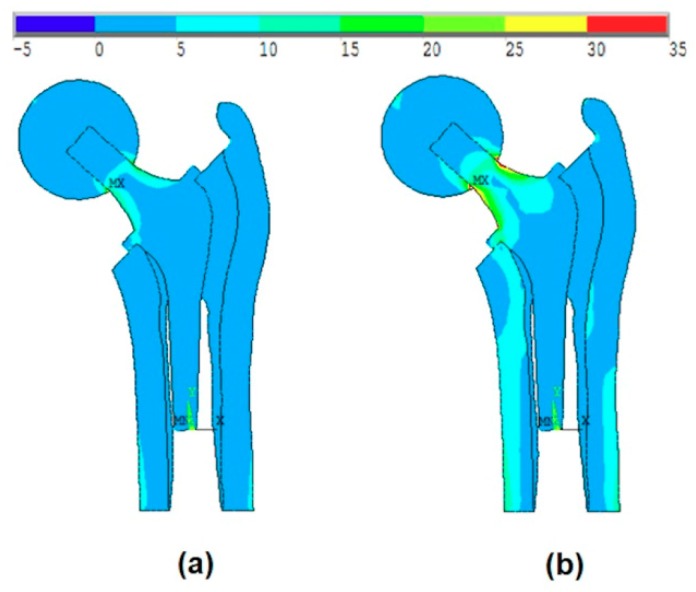
The effect of the introduction of the ball and bone anisotropy on the von-Mises stress distribution at (**a**) the mean point and (**b**) the MPP.

Case 2:

Figure 8a shows the effect of the introduction of the ball and bone anisotropy on the von-Mises stress distribution at the mean point, while Figure 8b shows the von-Mises stress distribution at the MPP.

The MPP is found on the failure scenario of the cancellous tissue in the transversal direction where the reliability index is β=4.38.

**Figure 9:**
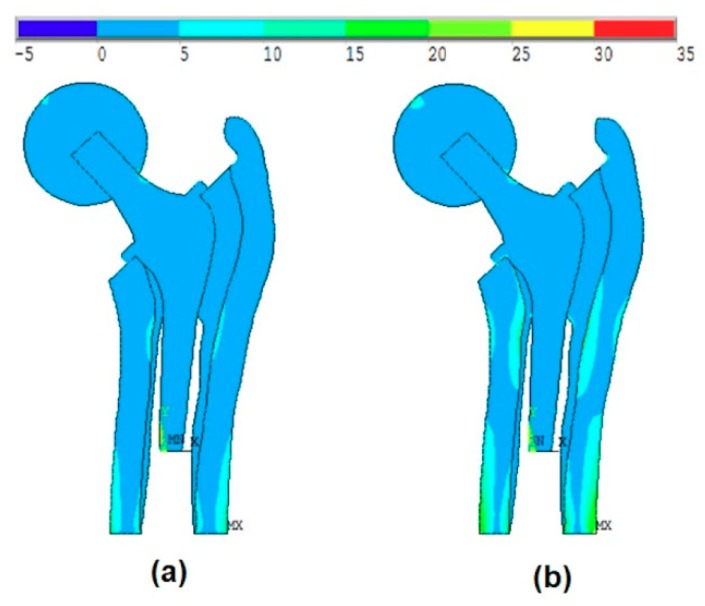
The effect of the introduction of the ball and bone anisotropy on the von-Mises stress distribution at (**a**) the mean point and (**b**) the MPP.

Case 3:

Figure 9a shows the effect of the introduction of the ball and bone anisotropy on the von-Mises stress distribution at the mean point, while Figure 9b shows the von-Mises stress distribution at the MPP.

The MPP is found on the failure scenario of the cancellous tissue in the transversal direction where the reliability index is β=2.93. Table 5 shows the input and output parameters of the mean point and the MPP for the three day-by-day loading cases (L1, L2, and L3). For the first and second loading cases (L1 and L2), the maximum stress value of the cancellous tissue in the transversal direction is almost equal to its yield stress value in the same direction ($\sigma_{\max}^{Can-Trans}$ ≈ $\sigma_{Yield}^{Can-Trans}$). However, for the third loading case L3, the maximum stress value of the cancellous tissue in the axial direction is almost equal to its yield stress value in the same direction ($\sigma_{\max}^{Can-Axial}$ ≈ $\sigma_{Yield}^{Can-Axial}$). At the MPP, there is no big difference between the resulting values of the elasticity modulus in the radial or circumferential directions, which confirms that the materials can be treated as transversal isotropic materials.

According to the Joint Committee on Structural Safety (JCSS) [28], the reliability index interval for structural engineering is β∈[3−4.25]. A detailed study of target safety indices can be found in Jeppsson [29]. Consequently, improving the reliability level is needed, as the reliability index of this interval has been found, β=2.93, in case 3 (L3). In this connection, the geometry can be optimized to meet the required reliability levels. The RBDO procedure is carried out to provide the reliability-based optimal geometry.

### 3.2. RBDO Results

The current configuration of the studied stem, especially for the third loading case (L3), is less reliable than the codes of practice, such as Eurocodes (European norms) and ACI (American norms) [30]. Thus, we need to modify the geometry of the stem to improve the reliability level. Here, the target reliability level is considered as β=3.8 (this target reliability index is used in structural engineering). Three design variables corresponding to stem dimensions are considered (A,B,C). The standard deviations are proposed proportional to the mean values (10%).

**Figure 10:**
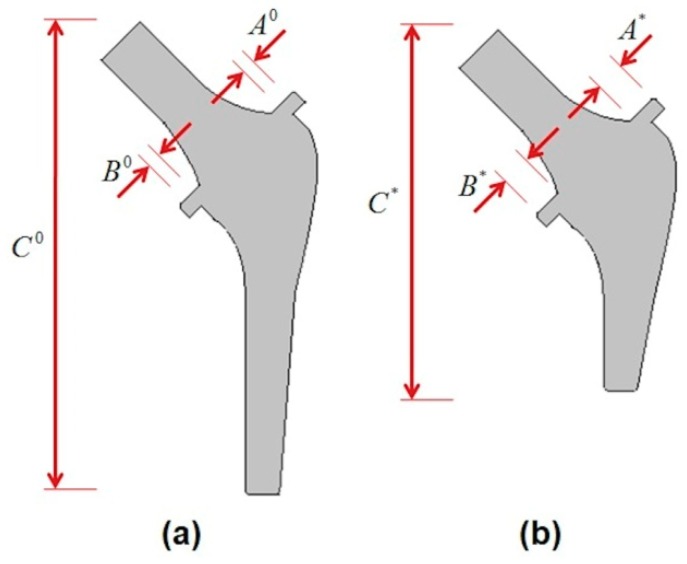
(**a**) Initial point and (**b**) optimal configuration.

After having performed the RBDO algorithm for the most critical loading case (L3), the resulting reliable configuration is shown in Figure 10, which corresponds to the required reliability index β=3.8. As shown in Table 6, the RBDO results verify the double failure modes ($\sigma_{\max}^{Can-Trans}$ ≈ $\sigma_{Yield}^{Can-Trans}$ and $\sigma_{\max}^{Can-Axial}$ ≈ $\sigma_{Yield}^{Can-Axial}$).

Therefore, the resulting RBDO configuration shown in Figure 10b respects the required reliability level for the most critical loading case L3. The initial and optimal stem dimensions are presented in Table 7.

**Figure 11:**
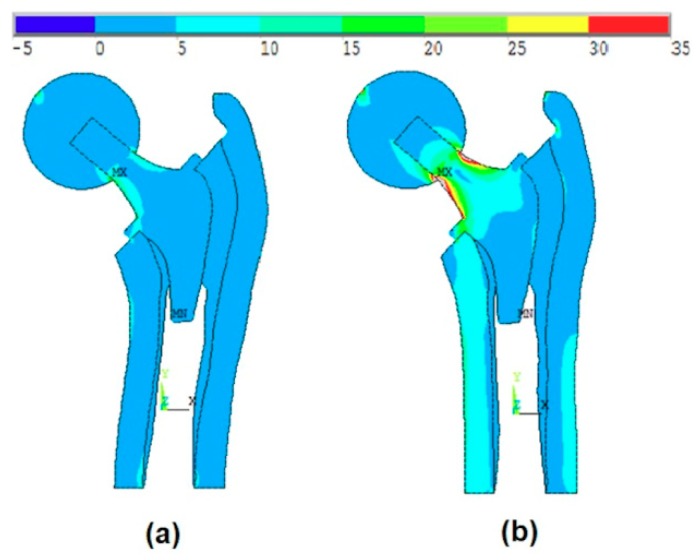
The effect of the introduction of the ball, bone anisotropy, and the stem shape optimization on the von-Mises stress distribution at (**a**) the optimal point and (**b**) the failure point.

In the following, the RBDO results of the three different loading cases L1, L2, and L3 are explained:

Case 1:

Figure 11a shows the effect of the introduction of the ball, bone anisotropy, and the stem shape optimization on the von-Mises stress distribution at the optimal point, while Figure 11b shows the von-Mises stress distribution at the failure point. The failure point is found on the failure scenario of the cancellous tissue in the transversal direction.

**Figure 12:**
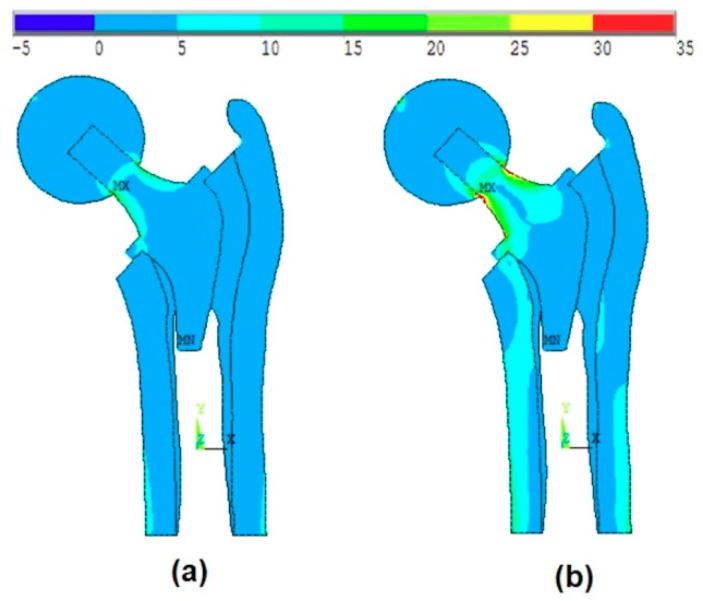
The effect of the introduction of the ball, bone anisotropy, and the stem shape optimization on the von-Mises stress distribution at (**a**) the optimal point and (**b**) the failure point.

Case 2:

Figure 12a shows the effect of the introduction of the ball, bone anisotropy, and the stem shape optimization on the von-Mises stress distribution at the mean point, while Figure 12b shows the von-Mises stress distribution at the failure point. The failure point is found on the failure scenario of the cancellous tissue in the transversal direction.

**Figure 13:**
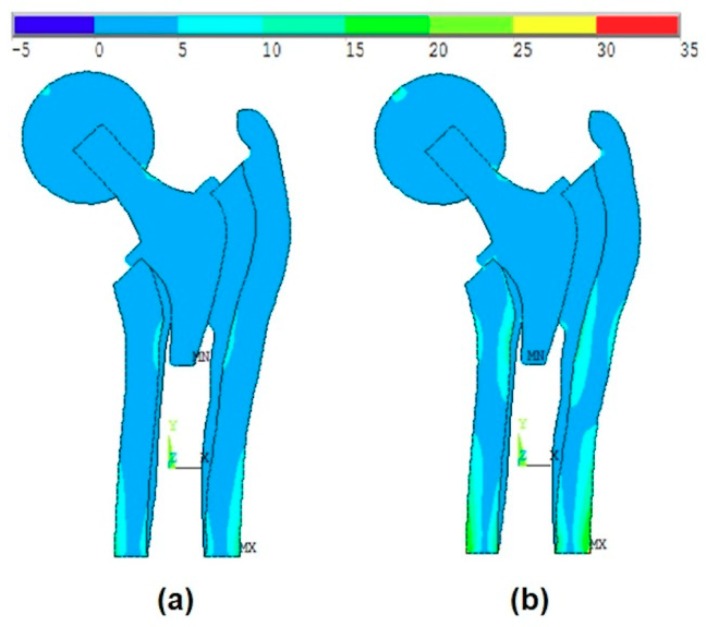
The effect of the introduction of the ball, bone anisotropy, and the stem shape optimization on the von-Mises stress distribution at (**a**) the optimal point and (**b**) the failure point.

Case 3:

Figure 13a shows the effect of the introduction of the ball, bone anisotropy, and the stem shape optimization on the von-Mises stress distribution at the mean point, while Figure 13b shows the von-Mises stress distribution at the failure point. The failure point is found on the failure scenario of the cancellous tissue in the axial direction.

When comparing the failure point and the MPP in Table 5 and Table 6, the results show almost the same failure limitations for all loading cases: $\sigma_{\max}^{Can-Trans}$ ≈ $\sigma_{Yield}^{Can-Trans}$, $\sigma_{\max}^{Can-Trans}$ ≈ $\sigma_{Yield}^{Can-Trans}$, and $\sigma_{\max}^{Can-Axial}$ ≈ $\sigma_{Yield}^{Can-Axial}$ for L1, L2, and L3, respectively.

## 4. Discussion

The different results can be discussed according to three aspects: Simulation, reliability, and reliability-based design optimization aspects. For the simulation aspect, when comparing the studied model to the previous works, two different changes can be noted: Geometry and material properties. In the works of Kharmanda [2] and Kharmanda et al. [15], the studied model was analyzed considering that the bone material properties were isotropic and the body forces were applied directly on the stem (without ball). Studying the stem without the existence of the ball would entirely change the study as the force values were extracted from Beaupré et al. [17] whom carried out their study on the femoral part of a real bone. In addition, in the works of Kharmanda et al. [11] and Kharmanda et al. [27], the studied models were analyzed considering only that the bone material properties were isotropic. The consideration of isotropic bone material properties may affect the accuracy of the study as there is a significant difference between the values of Young’s modulus in the axial and the radial directions.

In this work, the studied model is analyzed considering the ball and the anisotropy of bone material properties. The effect of the ball was much more remarkable than the anisotropy of the bone material properties. The von-Mises stress distribution was very different especially when it concerns the maximum stress values. Figure 7a and Figure 8a show that the maximum stress values were located at the intersection between the ball and the head of the stem, while when applying the body forces on the stem (without ball, as shown in Kharmanda [2]), these values were located on the neck of the stem. On the other hand, when considering the anisotropy of the bone material properties, there were small differences in the resulting von-Mises stresses on the different layers. The resulting values in Table 4 are compared to the studied models in Kharmanda et al. [11] and Kharmanda et al. [27]. Therefore, the role of the ball is much more important than the anisotropy during the simulation study.

For the reliability aspect, two categories of reliability strategies can be distinguished in the literature: Dystem reliability analysis and component reliability. When considering a single failure scenario, the reliability algorithm leads to a single index, while when several failure cases are considered, the reliability algorithm leads to several indices [31]. This way, the application of these classical system reliability algorithms leads to eight reliability indices considering six failure modes (scenarios): Two for the isotropic materials (ball and stem) and six for the anisotropic materials (cortical and cancellous tissues in transversal and axial directions). However, the developed algorithm directly provides the most critical reliability index in the system reliability problems. Equation (20) presents the different constrains $g_{i}(x_{i})\leq 0$ as inequality constraints while the most critical one is considered as a limit state function $H(u_{i},y_{i})=0$. In addition, when treating the system reliability problem using the classical algorithm [14], the resulting reliability indices may not take the correlation between the different failure scenarios into account, while the developed algorithm in this work simultaneously deals with the different constraints. An additional difference between the other structural reliability algorithms and the developed one is the integration of optimized formulations of the living tissues (cortical and cancellous), which leads to new boundaries at each iteration (new optimization variable space at each iteration). Here, accurate bone formulations for yield stress against the elasticity modulus relationship are needed to be integrated into the developed algorithm. In the literature, there were no bone formulations for the yield stress against the elasticity modulus relationship in the transversal isotropic cases. The existing formulations are Lotz’s formulations for the elasticity modulus against the density relationship and for the yield stress against the density relationship [19]. After having developed formulations 12, 13, 14, and 15 considering Lotz’s formulations 1, 2, 3, and 4 for cortical tissue, and 5, 6, 7, and 8 for cancellous tissue, all the developed formulations 12, 13, 14, and 15 were compared to experimental results for the axial and transversal directions. The results showed a big difference between Lotz’s formulations and the experimental data for cortical and cancellous bone tissues. Thus, there is a strong need to develop the new formulations providing the optimum fitness curves of the existing experimental results. Therefore, optimized formulations 16 and 17 were developed and compared to Lotz’s formulations. These optimized formulations showed a good accuracy relative to Lotz’s formulations (see Table 2, Table 3 and Table 4). In order to integrate these optimized formulations into the developed reliability algorithm, the ultimate tensile strength of bone tissue was considered as a fraction of the compression strength. Thus, formulations 18 and 19 were integrated into the reliability algorithm.

The developed reliability algorithm in this work deals with multiple failure modes, while the previous reliability algorithm in Kharmanda [2] treated a single failure mode. In addition, the anisotropy and geometry affect the probabilistic model such as the mean point, which is considered a starting point for the current developed reliability algorithm. The reliability results are much more realistic than the previous results that were presented in Kharmanda [2] because of the consideration of the anisotropy on the bone material properties, as well as the application of body forces on the ball instead of directly applying them to the stem, and the consideration of multiple failure modes. According to the obtained reliability results, the failure scenario for all cases was related to the cancellous tissue in the transversal and/or axial directions. In the first and second cases, L1 and L2, the failure was found in the transversal direction of the cancellous tissue, while in the third case L3, it was found in the axial direction of the cancellous tissue. The yield stress in the cortical and cancellous tissues is related to Young’s modulus values (Equations (18) and (19)). The maximum stress value should not exceed these yield stresses values. In Table 5, the maximum values of von-Mises stress were presented for the different parts. In addition, the maximum stress values in both transversal and axial directions were presented for the cortical and cancellous tissues. The minimum value of the resulting reliability index was β=2.93∉[3−4.25]. As it is less than the lowest value in the reliability index interval for structural engineering [29], there is a strong need to improve this value to meet the standard interval. Accordingly, a RBDO procedure was then implemented to provide the suitable design with a controlled reliability index. In addition to considering bone isotropic material and the non-existence of the ball, the used RBDO method in our previous work [15] was carried out using the hybrid method, which has several drawbacks concerning computing time, especially when dealing with multiple failure modes.

In order to overcome these drawbacks, the OSF method was efficiently used for several failure modes in this work. Here, three geometrical parameters concerning the stem were considered to be optimized in order to control the reliability level ($\beta_{t}=3.8$). The optimized stem is shown in Figure 7b and its corresponding dimensions are presented in Table 7. When performing a direct simulation of the optimal configuration and the failure point, it is exactly found that the same scenarios of failure of the MPP that are presented in Table 5 have been obtained. The resulting values are almost close to each other (see Table 5 and Table 6). For both points, the failure mode for the first and second loading cases occurs in the cancellous tissue on the transversal directions and for the third loading case in the cancellous tissue in the axial direction. This result confirms the robustness of the RBDO using the OSF for multiple failure scenarios. This study is limited to the developed 2D model, which was employed in order to reduce the computing time, as well as to guarantee convergence stability.

In general, the replaced hip prosthesis may have different outcomes according to several factors. These factors are, but not limited to, hip design, surgeon experience, and implantation technique. In addition to this, patient characteristics such as age, sex, weight, activity level, and overall health affect the choice of the replaced hip prosthesis type. One of the most clinical implications of this research is to reduce the risk of bone fracture as all failure modes introduced in this research focus on the maximum von-Mises stress values, which are considered fracture indicators [32]. Furthermore, the resulting RBDO solution can be classified as a short stem. This kind of stem could be utilized to ensure primary stability of hip prostheses [33].

## 5. Conclusions

The presented work generally consists of three stages: The first stage involves developing an efficient reliability algorithm that can be used to transfer a system reliability problem to a single-component reliability problem taking into account several failure modes. The second stage involves developing new bone formulations for the yield stress against the elasticity modulus relationship in the transversal isotropic case. In the third stage, the reliability-based design optimization using optimum safety factors is added in order to control the reliability level, in which the stem geometry is modified to meet the required reliability levels.

Finally, future work arising from this study includes the consideration of a 3D model with the objective to improve the realism of the solution provided. In order to perform such a realistic study, a large number of applied force data along with transient analyses are required to simulate the gait cycle. Several patients are needed to carry out this costly study (a large amount of data, lengthy computing time, and high computing cost). The different results show that the failure scenario may happen in the cancellous tissue. Therefore, this tissue should be treated as a porous material in order to obtain more realistic results.

## Figures and Tables

**Table 1:** Force components [10].

Loading Case	Body Forces		Muscle Forces	
	$F_{x}^{B}[N]$	$F_{y}^{B}[N]$	$F_{x}^{M}[N]$	$F_{y}^{M}[N]$
L1	942.41	−2116.68	−330.04	620.71
L2	−299.71	−1118.54	48.85	347.58
L3	1283.35	−865.63	−268.43	383.36

**Table 2:** A comparison of experimental compressive stress with Kharmanda’s and Lotz’s models in the axial direction.

Experimental Point Number		$P_{1}^{Cor-Axial}$	$P_{2}^{Cor-Axial}$	$P_{3}^{Cor-Axial}$
Authors		Keaveny et al. [12]	Gibbons [20]	Reilly et al. [21]
**Young’s Modulus (MPa)**		17,000	17,400	17,900
**Compressive Strength (MPa)**	**Experimental Results**	192	196	205
	**Kharmanda’s Model**	192.64	196.90	202.21
	**Lotz’s Model**	261.95	265.69	270.32

**Table 3:** A comparison of experimental compressive stress with the newly proposed and Lotz’s models in the transversal direction.

Experimental Point Number		$P_{1}^{Cor-Trans}$	$P_{2}^{Cor-Trans}$	$P_{3}^{Cor-Trans}$
Authors		Keaveny et al. [12]	Gibbons [20]	Reilly et al. [21]
**Young’s Modulus (MPa)**		10,100	11,700	12,000
**Compressive Strength (MPa)**	**Experimental Results**	131	135	137
	**Newly Proposed Model**	130.99	135.69	136.52
	**Lotz’s Model**	152.25	175.34	179.65

**Table 4:** A comparison of experimental compressive stress with Kharmanda’s and Lotz’s models for cancellous bone in axial and transversal directions.

Experimental Point Number		$P_{1}^{Can-Axial}$	$P_{2}^{Can-Trans}$
**Young’s Modulus (MPa)**		431	127
**Compressive Strength (MPa)**	**Experimental Results**	4.5	1.6
	**Kharmanda’s Model**	6.09	1.93
	**Lotz’s Model**	7.39	3.90

**Table 5:** Input and output parameters of the developed reliability algorithm.

Parameters	Mean Point			MPP		
	*L*1	*L*2	*L*3	*L*1	*L*2	*L*3
$F_{x}^{W}$	9.4241	2.9971	12.8335	0.40083	5.9822	18.093
$F_{y}^{W}$	21.1668	11.1854	8.6563	38.048	22.333	14.695
$F_{x}^{M}$	3.3004	0.4885	2.6843	0.03177	0.97505	0.40164
$F_{y}^{M}$	6.2071	3.4758	3.8336	11.83	6.934	5.5361
$E_{x}^{Cor}$	6300			6028.7	4437.8	4483.1
$E_{y}^{Cor}$	16,000			15,947	11,210	11,861
$E_{z}^{Cor}$	6300			6267.5	4866.7	5222
$\nu_{xy}^{Cor}$	0.3			0.33196	0.3533	0.35498
$\nu_{yz}^{Cor}$	0.3			0.32521	0.30673	0.33672
$\nu_{xz}^{Cor}$	0.45			0.50257	0.53058	0.49293
$G_{xy}^{Cor}$	3300			3047.9	3298	3166.8
$G_{yz}^{Cor}$	3300			2631	3572.9	2631.9
$G_{xz}^{Cor}$	3600			2787	3584	3494.9
$E_{x}^{Can}$	822			577.78	575.9	622.13
$E_{y}^{Can}$	1352			1346.6	947.22	957.3
$E_{z}^{Can}$	822			813.01	577.68	770.78
$\nu_{xy}^{Can}$	0.3			0.34892	0.35957	0.3127
$\nu_{yz}^{Can}$	0.3			0.31845	0.35887	0.34197
$\nu_{xz}^{Can}$	0.3			0.34371	0.3438	0.31825
$G_{xy}^{Can}$	399			284.42	279.54	369.95
$G_{yz}^{Can}$	399			341.8	289.64	339.07
$G_{xz}^{Can}$	370			262.5	369.05	259.89
β	----			3.91	4.38	2.93
$\sigma_{\max}^{Cor-VM}$	13.80	8.94	16.83	27.69	20.19	31.88
$\sigma_{\max}^{Cor-Trans}$	3.39	4.73	4.14	13.20	12.50	9.41
$\sigma_{Yield}^{Cor-Trans}$	58.48			57.87	53.76	53.89
$\sigma_{\max}^{Cor-Axial}$	9.96	8.32	15.79	23.63	17.99	29.39
$\sigma_{Yield}^{Cor-Axial}$	90.98			90.70	65.12	68.67
$\sigma_{\max}^{Can-VM}$	4.24	3.21	5.59	7.69	5.94	9.20
$\sigma_{\max}^{Can-Trans}$	1.27	2.70	1.67	**5.60**	**5.47**	2.91
$\sigma_{Yield}^{Can-Trans}$	7.82			**5.61**	**5.60**	6.02
$\sigma_{\max}^{Can-Axial}$	3.47	1.89	5.52	3.13	3.81	**9.00**
$\sigma_{Yield}^{Can-Axial}$	12.48			12.44	8.93	**9.02**
$\sigma_{\max}^{Stem-VM}$	17.74	18.38	7.33	50.55	36.69	10.08
$\sigma_{\max}^{Ball-VM}$	17.74	18.38	9.32	50.55	36.69	13.77

**Table 6:** Output parameters of the RBDO algorithm.

Parameters	Optimum Solution			Design Point		
	*L*1	*L*2	*L*3	*L*1	*L*2	*L*3
$\sigma_{\max}^{Cor-VM}$	14.61	10.29	16.91	32.31	20.33	32.07
$\sigma_{\max}^{Cor-Trans}$	4.85	4.77	4.38	13.42	12.56	9.42
$\sigma_{Yield}^{Cor-Trans}$	58.48			57.87	53.76	53.89
$\sigma_{\max}^{Cor-Axial}$	12.80	8.78	15.87	28.93	18.11	29.53
$\sigma_{Yield}^{Cor-Axial}$	90.98			90.70	65.12	68.67
$\sigma_{\max}^{Can-VM}$	4.26	3.05	5.56	7.38	5.57	9.25
$\sigma_{\max}^{Can-Trans}$	1.24	2.80	1.70	**5.79**	**5.69**	2.95
$\sigma_{Yield}^{Can-Trans}$	7.82			**5.61**	**5.60**	6.02
$\sigma_{\max}^{Can-Axial}$	3.44	1.90	5.50	3.12	3.83	**9.03**
$\sigma_{Yield}^{Can-Axial}$	12.48			12.43	8.93	**9.02**
$\sigma_{\max}^{Stem-VM}$	18.62	19.26	8.02	53.01	38.46	10.09
$\sigma_{\max}^{Ball-VM}$	18.62	19.26	11.27	53.01	38.46	16.95

**Table 7:** Initial and optimal stem dimensions.

Parameters	*A*	*B*	*C*
**Initial Point**	4.00	3.95	114.33
**Optimal Point**	4.36	4.32	87.35
